# Supplementary figures and images for: Epstein-Barr virus latent membrane protein 1 subverts IMPDH pathways to drive B-cell oncometabolism
Source: PLoS Pathog. 2025 May 14;21(5):e1013092. doi: 10.1371/journal.ppat.1013092 (PMC12169587; doi:10.1371/journal.ppat.1013092)

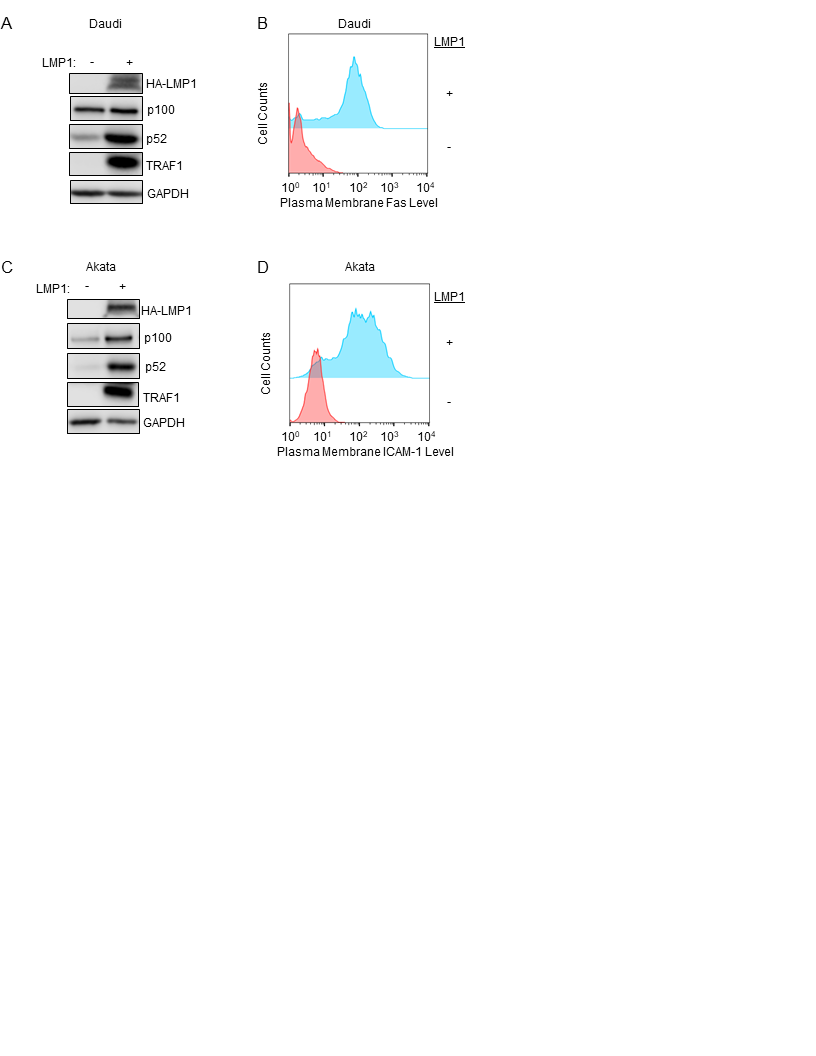

Supplement: S1 Fig — (A) Immunoblot analysis of whole cell lysates (WCL) from Daudi cells mock induced or induced for LMP1 expression by doxycycline (250ng/mL) for 24 hours. LMP1 expression induced non-canonical NF-κB activity, as judged by processing of the p100 precursor into the p52 subunit, and induced expression of the well characterized LMP1/NF-κB target TRAF1. (B) FACS analysis of plasma membrane Fas abundance in Daudi cells mock induced or induced for LMP1 expression for 24 hours, as in (A). Conditional LMP1 expression highly induced expression of the well characterized LMP1 target Fas. (C) Immunoblot analysis of WCL from Akata cells mock induced or induced for LMP1 expression as in (A), indicating LMP1 induction of non-canonical NF-κB activity and LMP1 target TRAF1 expression. (D) FACS analysis of plasma membrane ICAM-1 abundance in Akata cells mock induced or induced for LMP1 expression as in (A). ICAM-1 rather than Fas was analyzed as basal Fas expression is aberrantly elevated in Akata cells. (TIF) [file ppat.1013092.s004.tif]

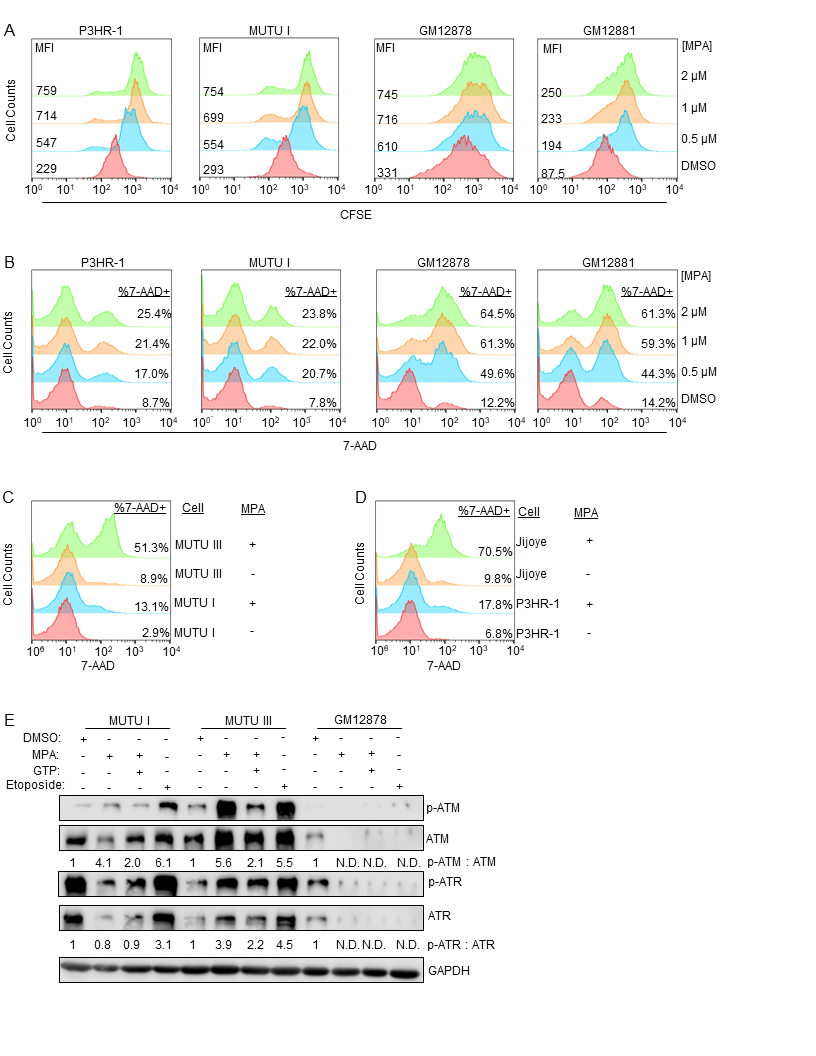

Supplement: S2 Fig — (A) FACS analysis of CFSE levels following 96 hours of treatment with the indicated MPA dosages in the indicated cell lines. (B) FACS analysis of 7-AAD uptake following 48 hours of treatment of indicated cell lines with the indicated MPA dosages. (C) FACS analysis of MUTU I versus III 7-AAD uptake following 48 hours of treatment with the indicated MPA dosages. (D) FACS analysis of P3HR-1 versus Jijoye cell 7-AAD uptake following 48 hours of treatment of MUTUI vs III with the indicated MPA dosages. (E) Immunoblot analysis of WCL from MUTU I, III or GM12878 treated with DMSO or 1 μM MPA with or without 100 μM GTP rescue for 48 hours. Cells treated with 50 μM etoposide as a control for ATM and ATR phosphorylation are included. Shown are the normalized phospho-ATM:ATM and phospho-ATR:ATR ratios calculated by densitometry analysis, with values in DMSO-treated cells set to 1. N.D., not determined. (TIF) [file ppat.1013092.s005.tif]

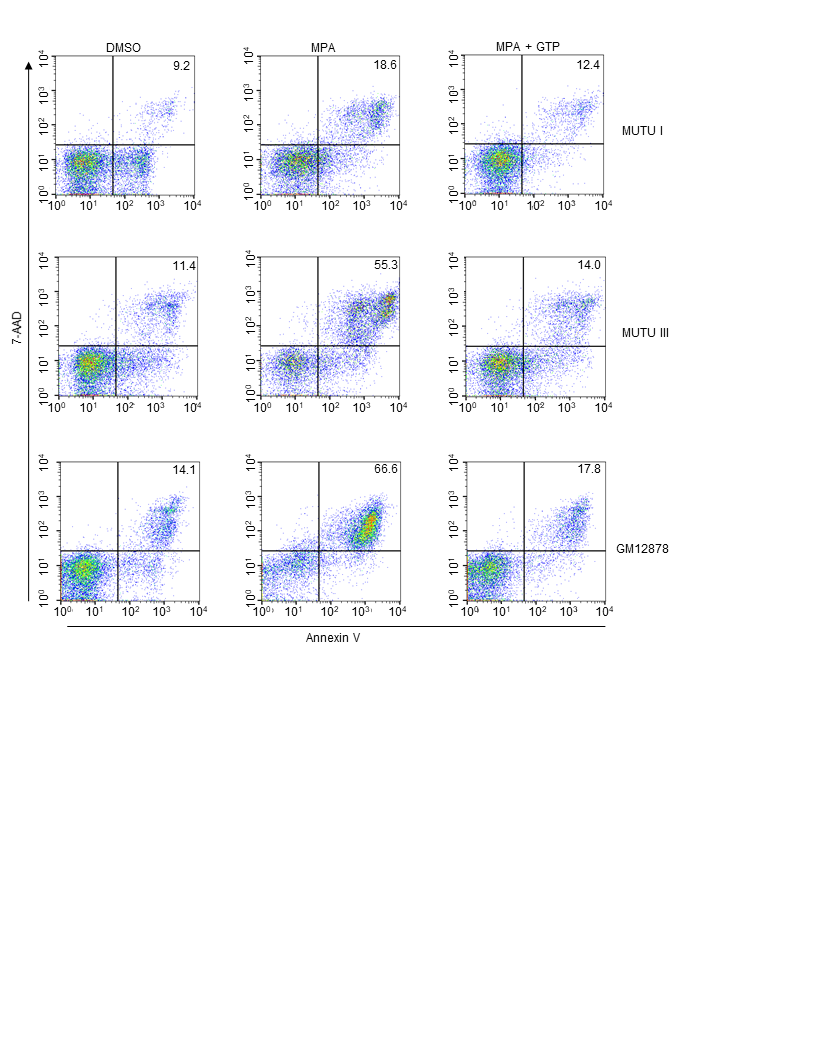

Supplement: S3 Fig — Shown are representative FACS plots from n = 3 replicates of the indicated cell lines treated with DMSO, 1 μM MPA or 1 μM MPA + 100 μM GTP for 48 hours, as shown in Fig 2F. (TIF) [file ppat.1013092.s006.tif]

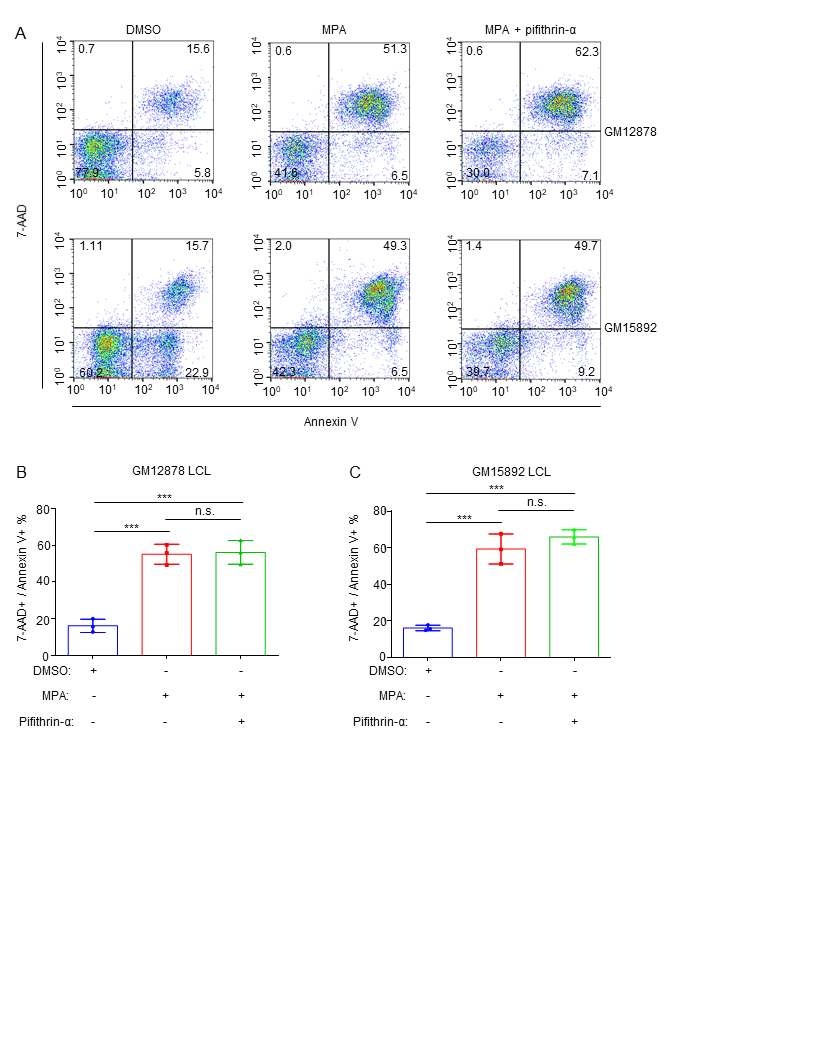

Supplement: S4 Fig — (A) Representative FACS plots from n = 3 replicates of GM15892 or GM12878 cells treated with DMSO vehicle, 1 μM MPA and/or 10 μM pifithrin-α for 48 hours, as indicated. (B and C) Mean ± SD percentages of double 7-AAD+ /Annexin V+ GM12878 (B) or GM15892 (C) treated with DMSO, MPA and/or pifithrin-α as in (A). (TIF) [file ppat.1013092.s007.tif]

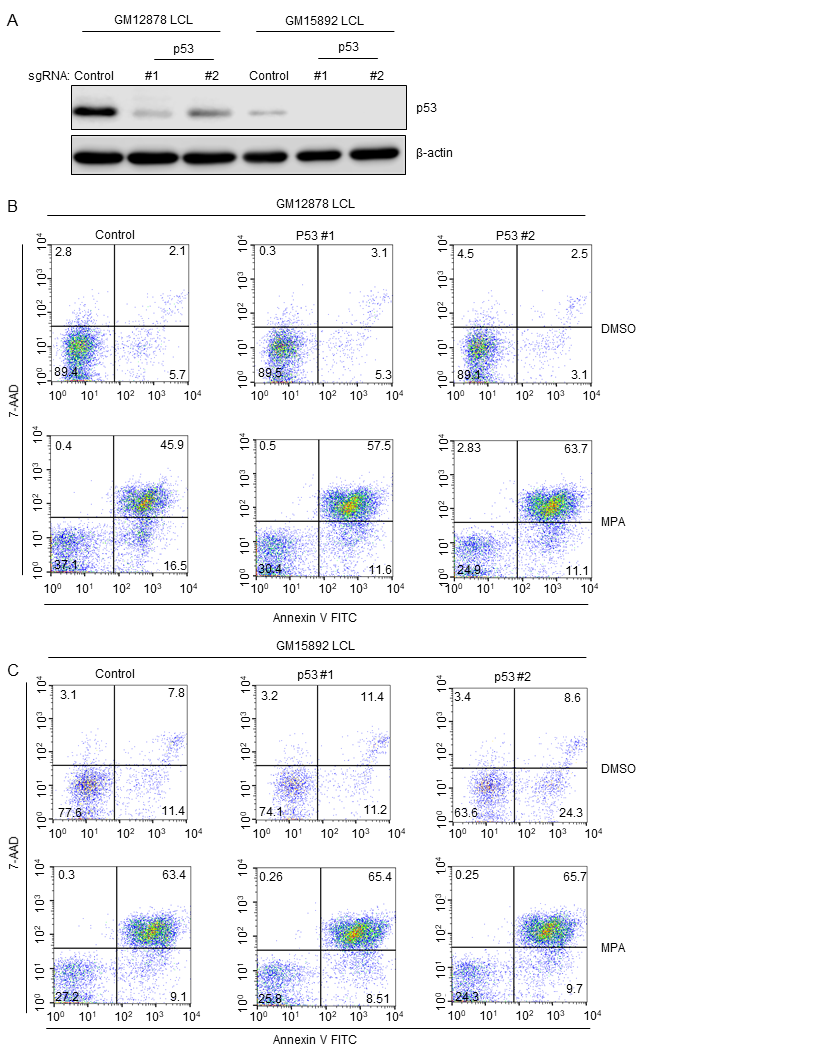

Supplement: S5 Fig — (A) Immunoblot analysis of CRISPR p53 editing in GM12878 and GM15892 LCLs. Shown are representative immunoblots from n = 3 replicates of WCL from LCLs expressing the indicated control or p53-targeting sgRNA. (B and C) Representative FACS plots of 7-AAD+ /Annexin V+ stained GM12878 (B) or GM15892 cells (C) transduced with control or p53 targeting single guide RNAs (sgRNA) from n = 3 replicates. Cells transduced with lentiviruses expressing the indicated sgRNAs, puromycin selected for four days and then treated with DMSO or MPA, as indicated for 48 hours. (TIF) [file ppat.1013092.s008.tif]

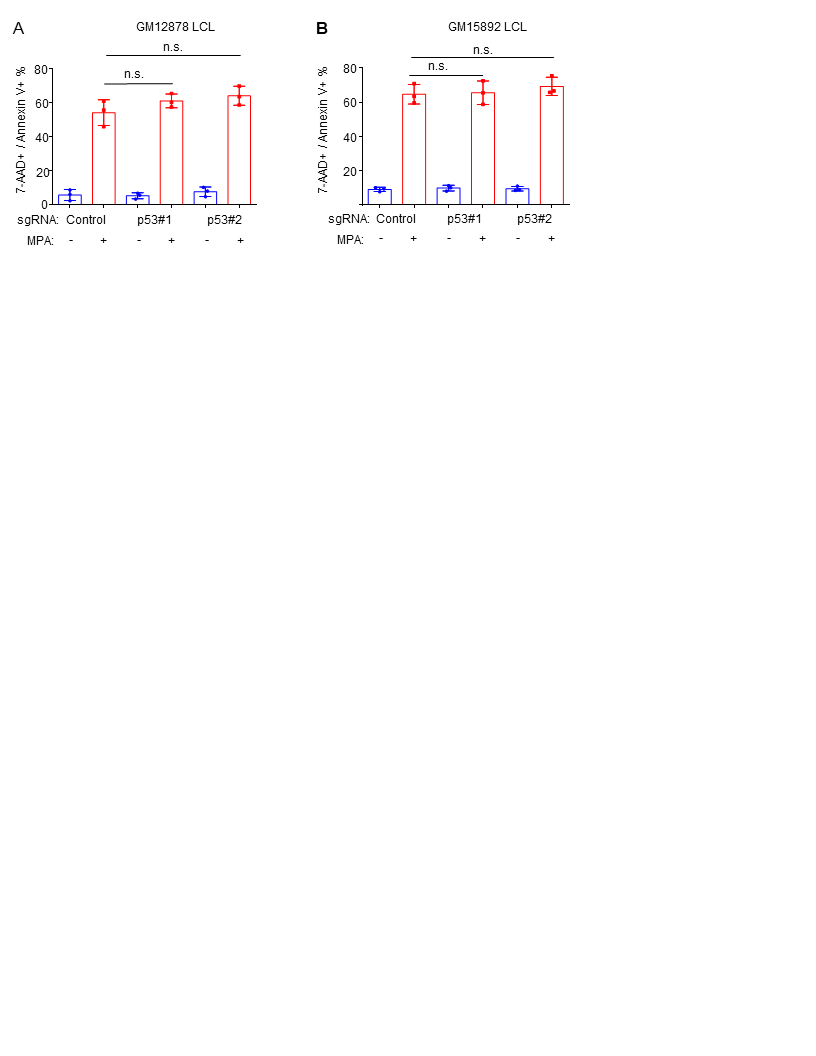

Supplement: S6 Fig — (A and B) Mean ± SD 7-AAD+/Annexin V+ from n = 3 replicates of Cas9 + GM12878 (A) or GM15892 (B) expressing the indicated control or p53 targeting sgRNA, as in S5 Fig. FACS 7-AAD+/Annexin V+ analyses were performed after 48 hours of treatment. (TIF) [file ppat.1013092.s009.tif]

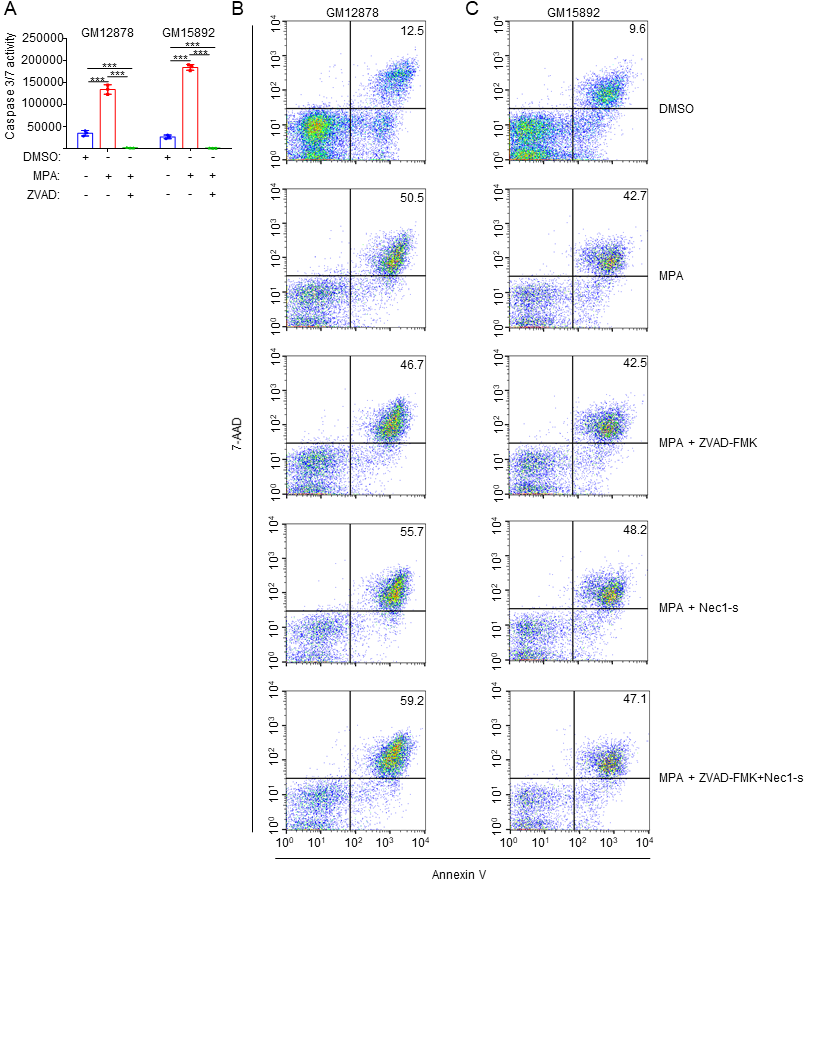

Supplement: S7 Fig — (A) Caspase 3/7 activity assays. Shown are mean ± SD Caspase 3/7 Glo values from n = 3 replicates of GM12878 and GM15892 LCLs treated with DMSO, MPA (1 μM) and/or the pan-caspase inhibitor ZVAD-FMK (40 μM) for 48 hours, as indicated. (B and C) GM12878 (B) or GM15892 (C) LCLs were treated with DMSO vehicle or with MPA (1 μM), the pan-caspase inhibitor ZVAD-FMK (40 μM), and/or the necroptosis pathway RIP1 kinase inhibitor Necrostatin-1s (20 μM) as indicated. FACS analysis of 7-AAD uptake and annexin V positivity was performed 48 hours later. Representative FACS plots from n = 3 independent replicates are shown. (TIF) [file ppat.1013092.s010.tif]

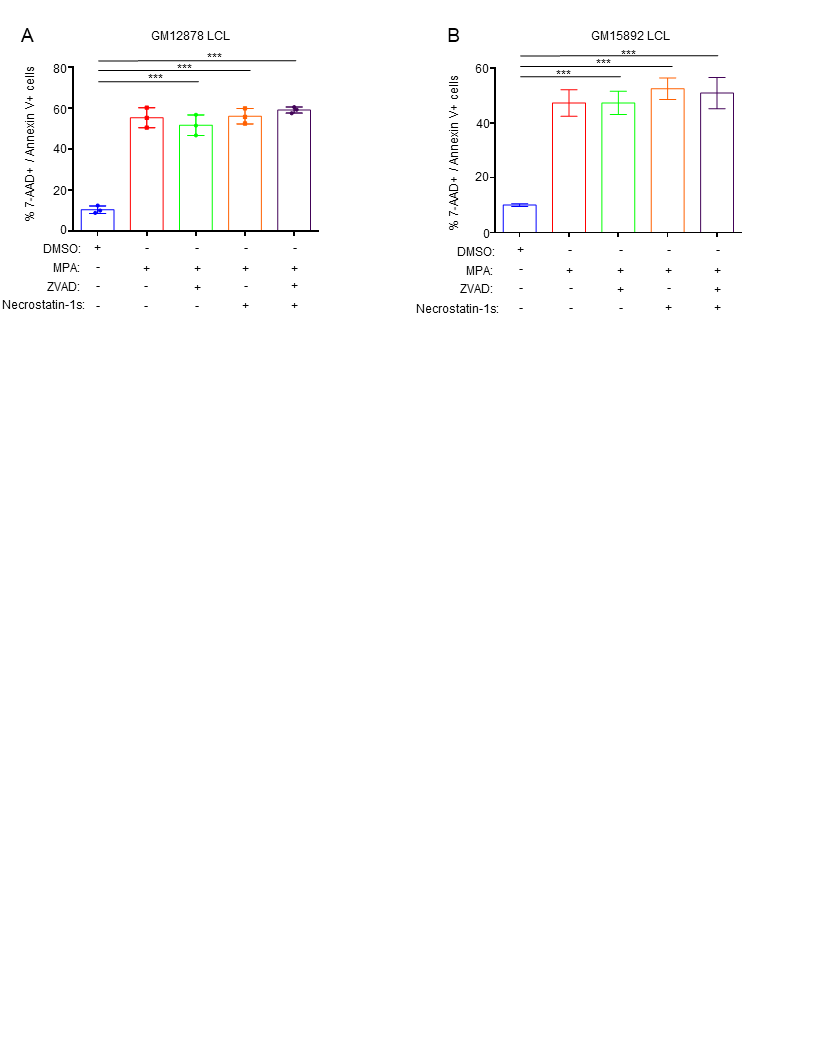

Supplement: S8 Fig — (A and B) GM12878 (A) and GM15892 (B) LCLs were treated with DMSO, MPA (1 μM), ZVAD-FMK (40 μM), and/or Necrostatin-1s (20 μM), as in S7 Fig. Shown are mean ± 7-AAD uptake values from n = 3 replicates. (TIF) [file ppat.1013092.s011.tif]

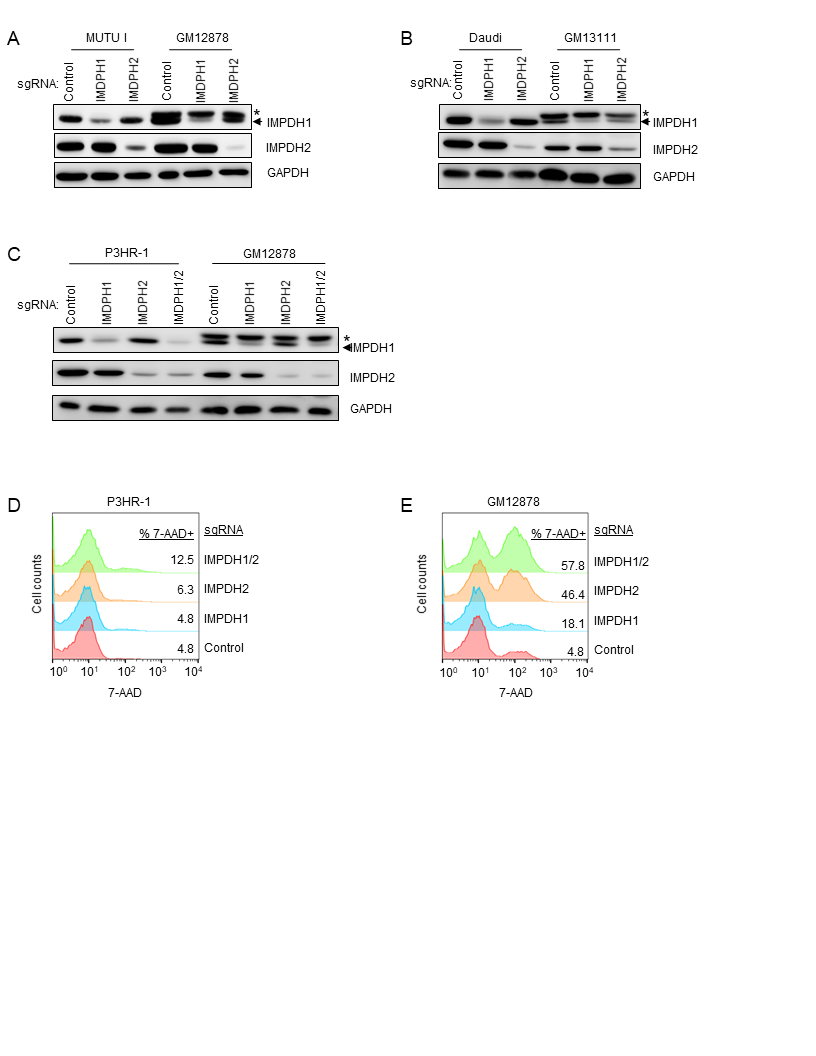

Supplement: S9 Fig — (A) Immunoblot analysis of IMPDH1 or IMPDH2 depletion. WCL from Cas9 + MUTU I or GM12878 expressing were subjected to immunoblot analysis at the indicated sgRNA at two days post-puromycin selection of successfully transduced cells. * = non-specific band. (B) Immunoblot analysis of IMPDH1 or IMPDH2 depletion in Daudi versus GM13111 WCL, as in (A). (C) Immunoblot analysis of IMPDH1 or IMPDH2 depletion in P3HR-1 versus GM12878 WCL, as in (A). (D) FACS analysis of 7-AAD uptake in Cas9 + P3HR-1 expressing the indicated sgRNA at 8 days post-puromycin selection of cells transduced by sgRNA expressing lentivirus. (E) FACS analysis of 7-AAD uptake in Cas9 + GM12878, as in (D). FACS plots and immunoblots are representative of n = 3 independent replicates. (TIF) [file ppat.1013092.s012.tif]

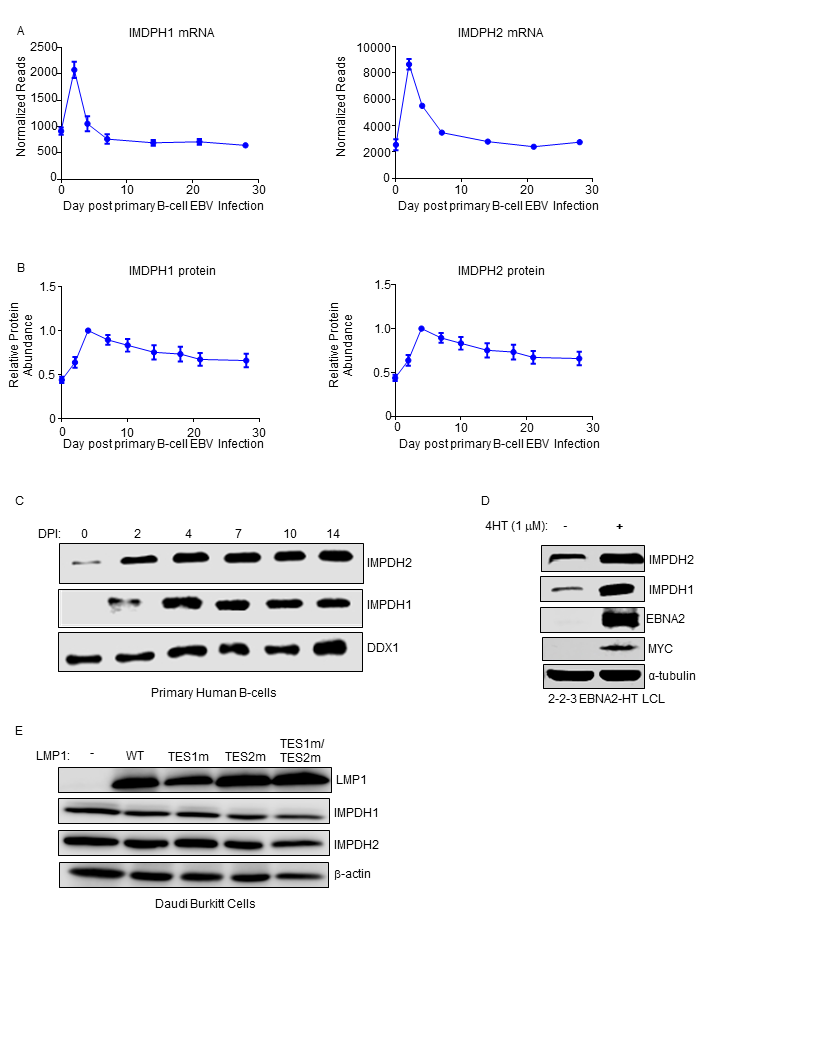

Supplement: S10 Fig — (A) Normalized IMPDH1 (left) versus IMPDH2 (right) reads from RNAseq analysis of primary human B cells at the indicated days post-infection by the EBV B95.8 strain. Shown are mean ± SD values from n = 3 replicates [42,63–65]. (B) Relative IMPDH1 (left) versus IMPDH2 (right) protein abundances from tandem mass tagged multiplexed mass spectrometry proteomic analysis of primary human B-cells at the indicated days post-infection by the EBV B95.8 strain. Shown are mean ± SD values from n = 4 replicates [42]. (C) Immunoblot analysis of WCL from primary human B-cells infected by B95.8 EBV at the indicated days post infection (DPI). DDX1 was used as a load control as its levels remain relatively unchanged between uninfected versus infected primary B-cells [42]. (D) Immunoblot analysis of WCL from 2-2-3 EBNA2-HT LCLs, which harbor a conditional EBNA2 allele that is fused to a modified estrogen receptor ligand binding domain, whose activity requires the presence of 4-hydroxytamoxifen (4-HT). WCL were generated at 48 hours post EBNA2 inactivation by 4-HT washout. (E) Immunoblot analysis of WCL from Daudi cells mock induced or induced for wildtype, TES1 point mutant (TES1m), TES2 point mutant (TES2m), or double TES1m/TES2m LMP1 for 24 hours by 250 ng/mL doxycycline. Immunoblots are representative of n = 3 independent replicates. (TIF) [file ppat.1013092.s013.tif]

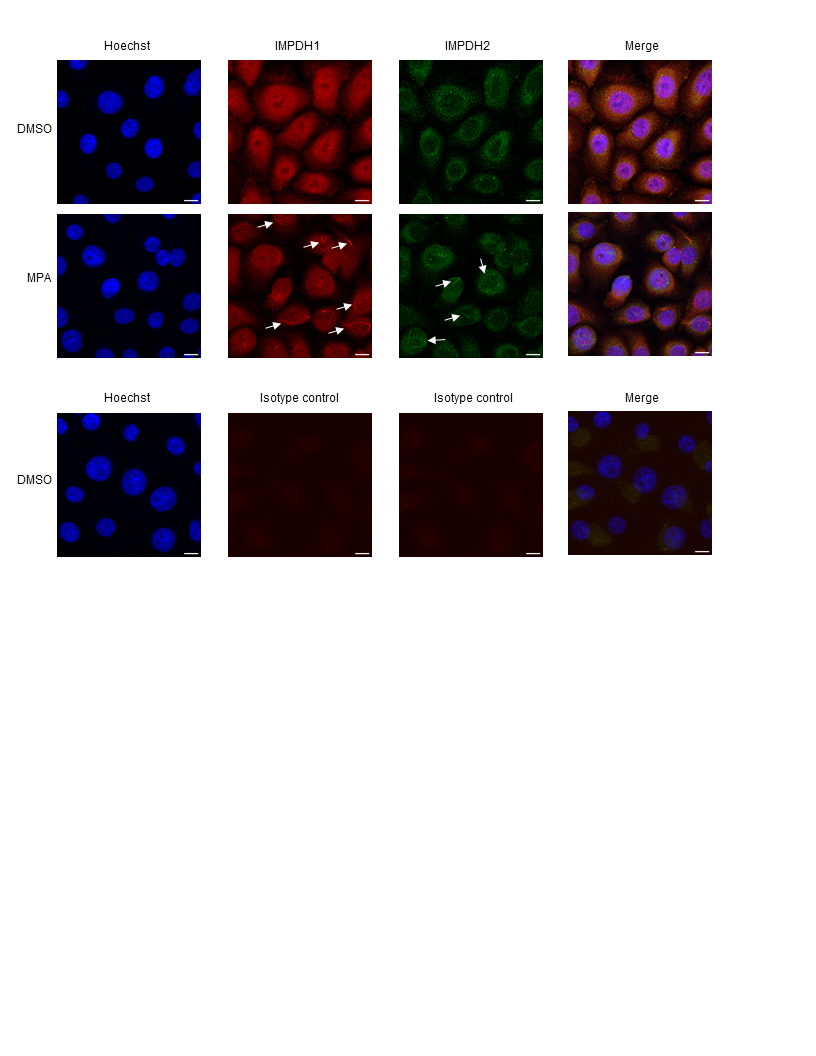

Supplement: S11 Fig — Representative images of normal oral keratinocytes treated with DMSO or with MPA (10 μM) for 24 hours and then stained for IMPDH1, IMPDH2 or with the Hoechst 33258 nuclear dye. Composite Z-stack images are also shown. Cells stained with antibody isotype controls are shown at bottom. Images are representative on n = 3 replicates. White scalebar indicates 10 μM distance. (TIF) [file ppat.1013092.s014.tif]

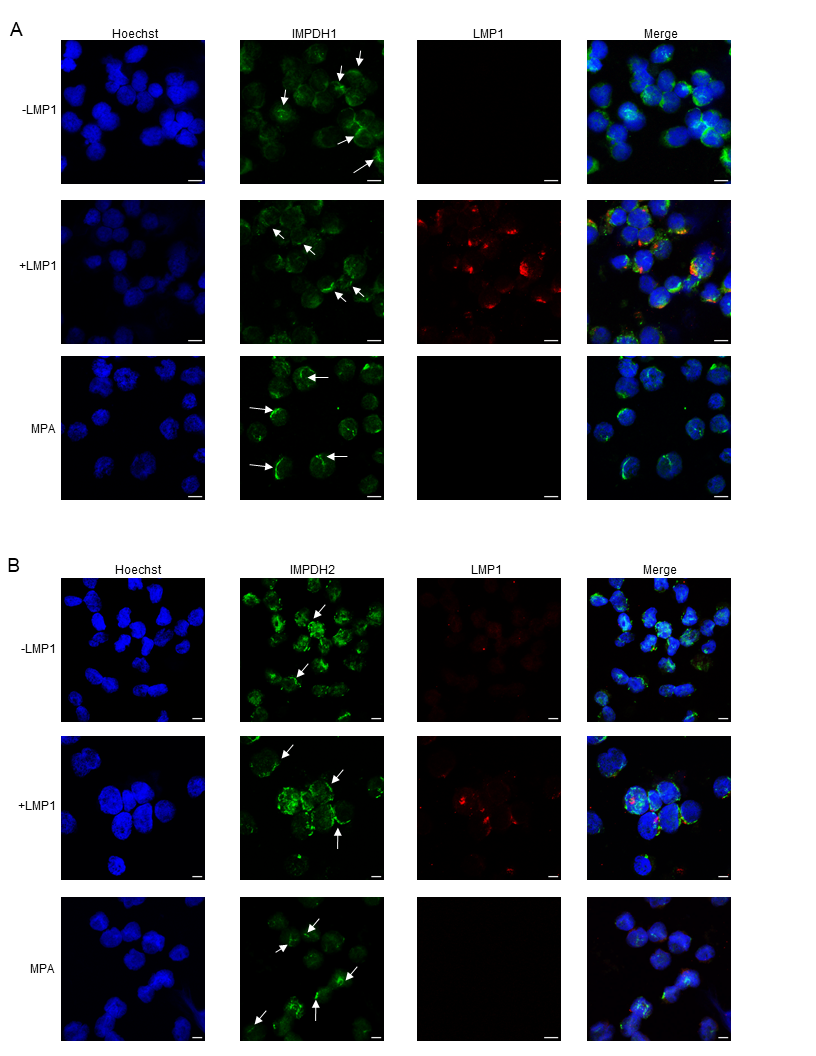

Supplement: S12 Fig — (A and B) Representative images from n = 3 replicates of Daudi cells mock-induced or induced for LMP1 expression for 24 hours or treated with MPA (1 μM) for 24 hours, stained and analyzed by confocal microscopy. (A) Cells were stained with αIMPDH1 and αHA-LMP1. (B) Cells were stained with αIMPDH2 and αHA-LMP1. White arrows indicate cytoophidium. White scalebar indicates 10 μM distance. (TIF) [file ppat.1013092.s015.tif]

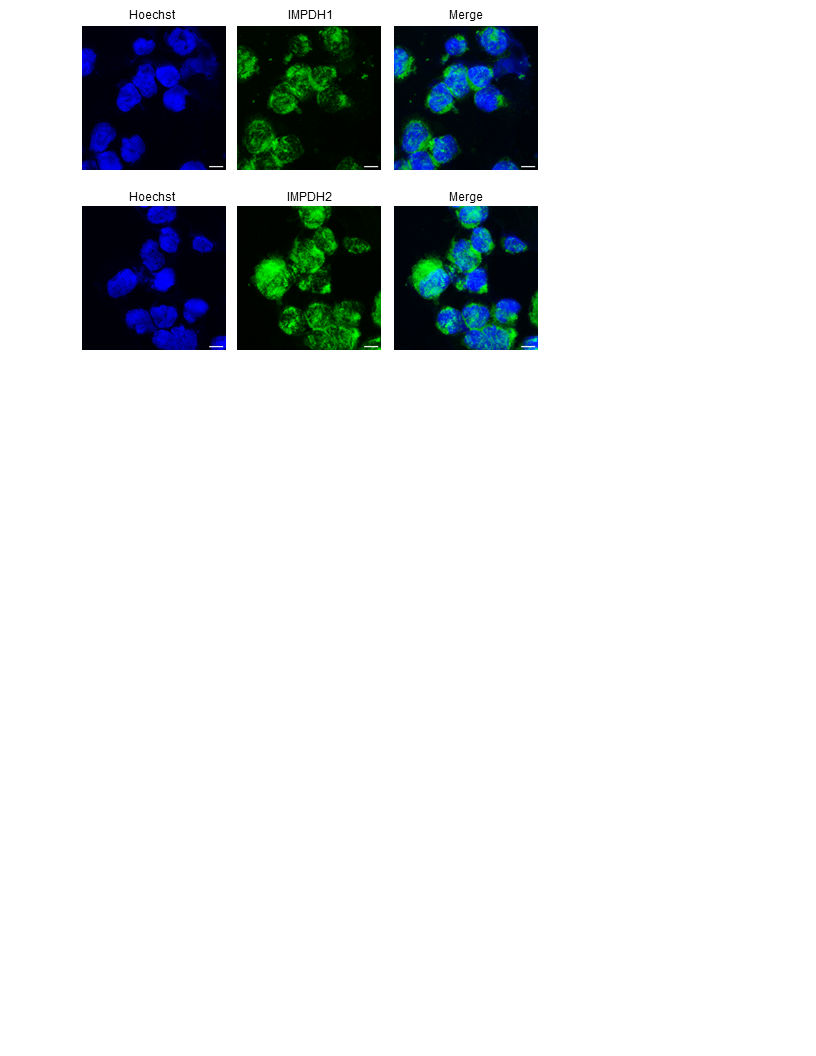

Supplement: S13 Fig — Representative images of GM12878 stained by Hoechst, anti-IMPDH1 or IMPDH2 and analyzed by confocal microscopy. White scalebar indicates 10 μM distance. (TIF) [file ppat.1013092.s016.tif]

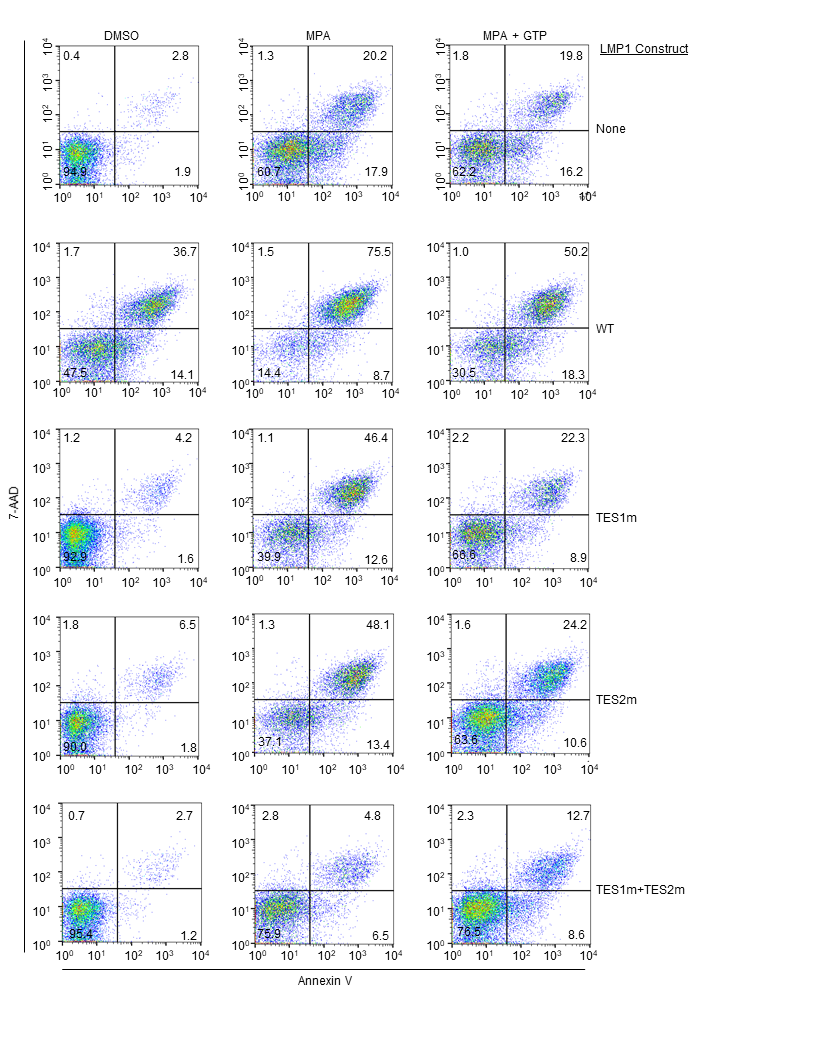

Supplement: S14 Fig — Representative FACS plots from n = 3 replicates of Daudi cells mock induced or induced for WT, TES1m, TES2m or TES1m+TES2m LMP1 expression for 24 hours and then treated with 1 μM MPA ± 100 μM GTP for 96 hours as indicated and as in Fig 4. Shown are FACS analysis of 7-AAD uptake versus Annexin V positivity. (TIF) [file ppat.1013092.s017.tif]

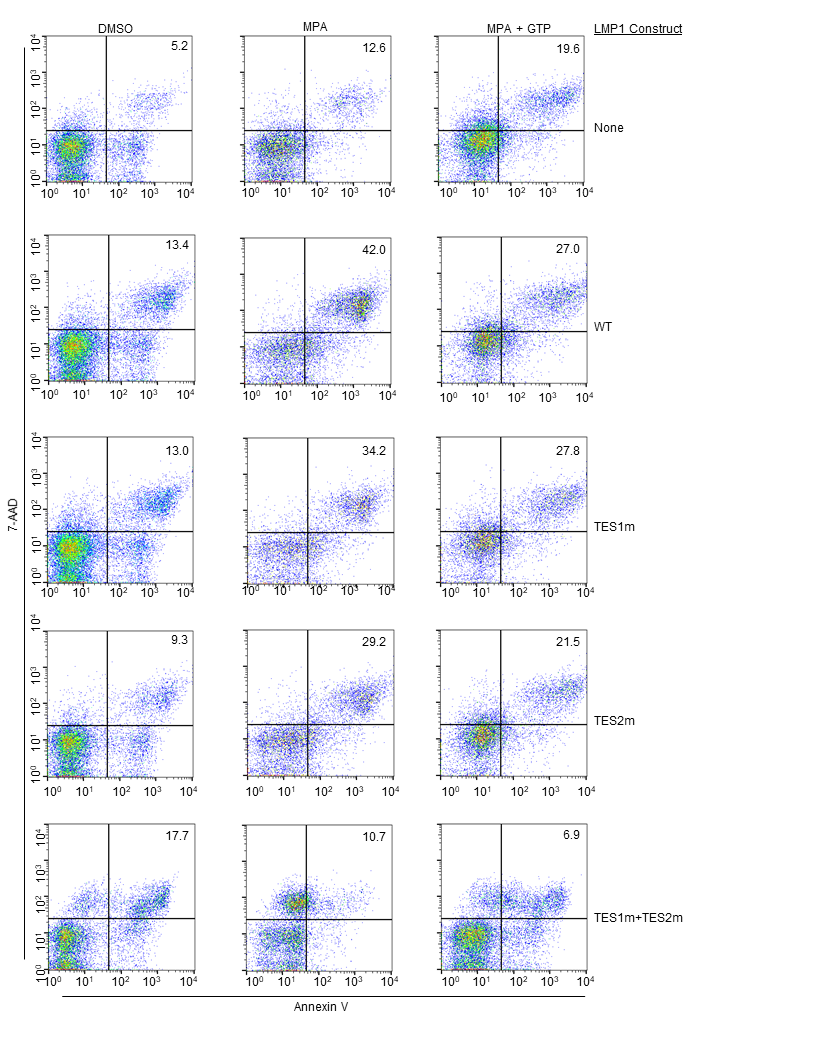

Supplement: S15 Fig — Representative FACS plots from n = 3 replicates of BL41 cells mock induced or induced for WT, TES1m, TES2m or TES1m+TES2m LMP1 expression for 24 hours and then treated with 1 μM MPA ± 100 μM GTP for 96 hours as indicated. Shown are FACS analysis of 7-AAD uptake versus Annexin V positivity. (TIF) [file ppat.1013092.s018.tif]

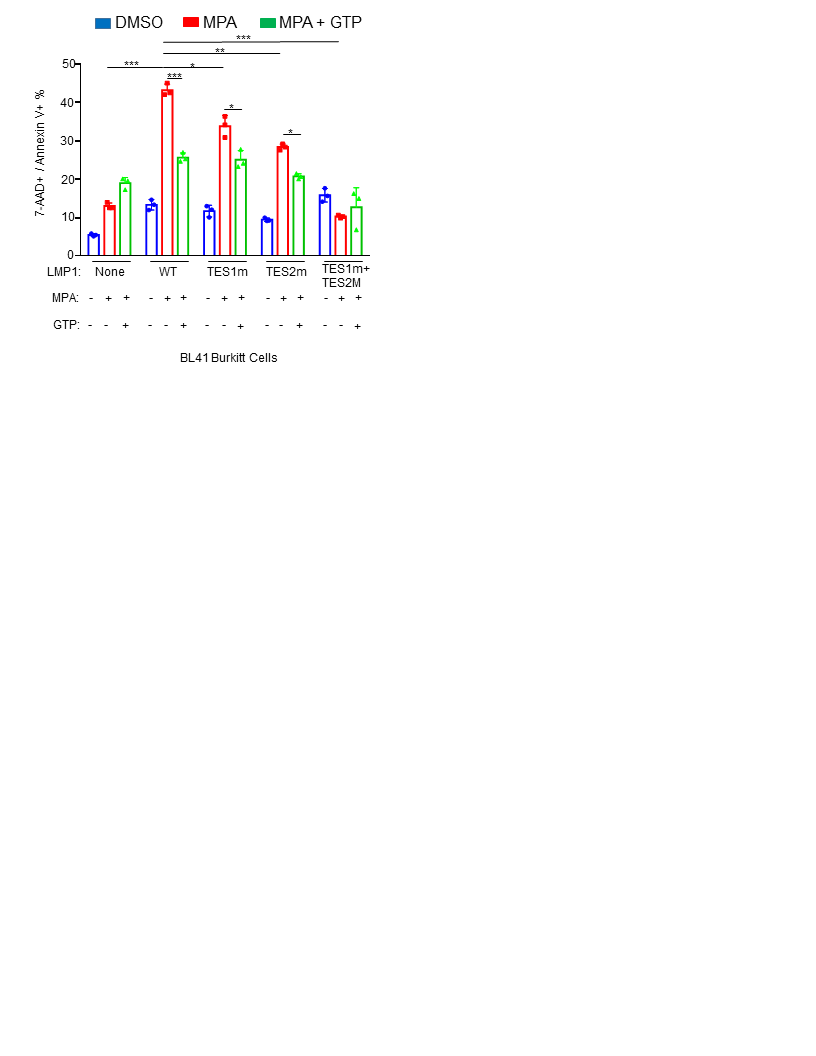

Supplement: S16 Fig — Mean ± SD percentages from n = 3 replicates from n = 3 replicates as in S15 Fig of BL41 cells mock induced or induced for the indicated LMP1 construct for 24 hours and then treated with DMSO or 1 μM MPA ±100 μM GTP for 96 hours. *P < 0.05, **P < 0.01, ***P < 0.005. (TIF) [file ppat.1013092.s019.tif]

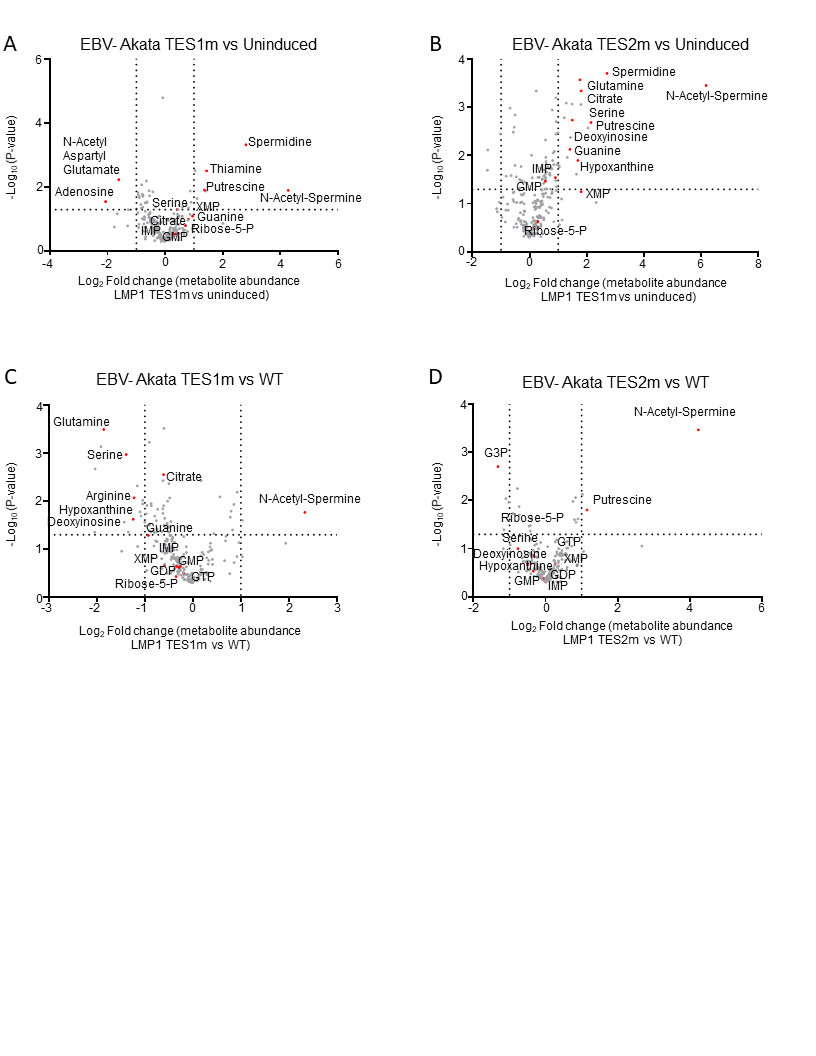

Supplement: S17 Fig — (A) Volcano plot of LC-MS metabolomic analysis of n = 6 replicates of EBV-negative Akata cells mock induced or doxycycline induced for LMP1 TES1m expression for 24 hours. Metabolites with higher abundance in LMP1 TES1 + cells have positive fold change values, whereas those higher in mock induced cells have negative fold change values. Selected metabolites are highlighted by red circles and annotated. (B) Volcano plot of LC-MS metabolomic analysis of n = 6 replicates of EBV-negative Akata cells mock induced or doxycycline induced for LMP1 TES2m expression for 24 hours, with selected metabolites highlighted as in (A). (C) Volcano plot of LC-MS metabolomic analysis of n = 6 replicates of EBV-negative Akata cells doxycycline induced for TES1m vs WT LMP1 expression for 24 hours, with selected metabolites highlighted. Replicates for this cross-comparison were induced side by side, prepared for and analyzed by LC-MS together on the same day to minimize batch effects. (D) Volcano plot of LC-MS metabolomic analysis of n = 6 replicates of EBV-negative Akata cells doxycycline induced for TES2m vs WT LMP1 expression for 24 hours, with selected metabolites highlighted. Replicates for this cross-comparison were induced side by side, prepared for and analyzed by LC-MS together on the same day to minimize batch effects. (TIF) [file ppat.1013092.s020.tif]

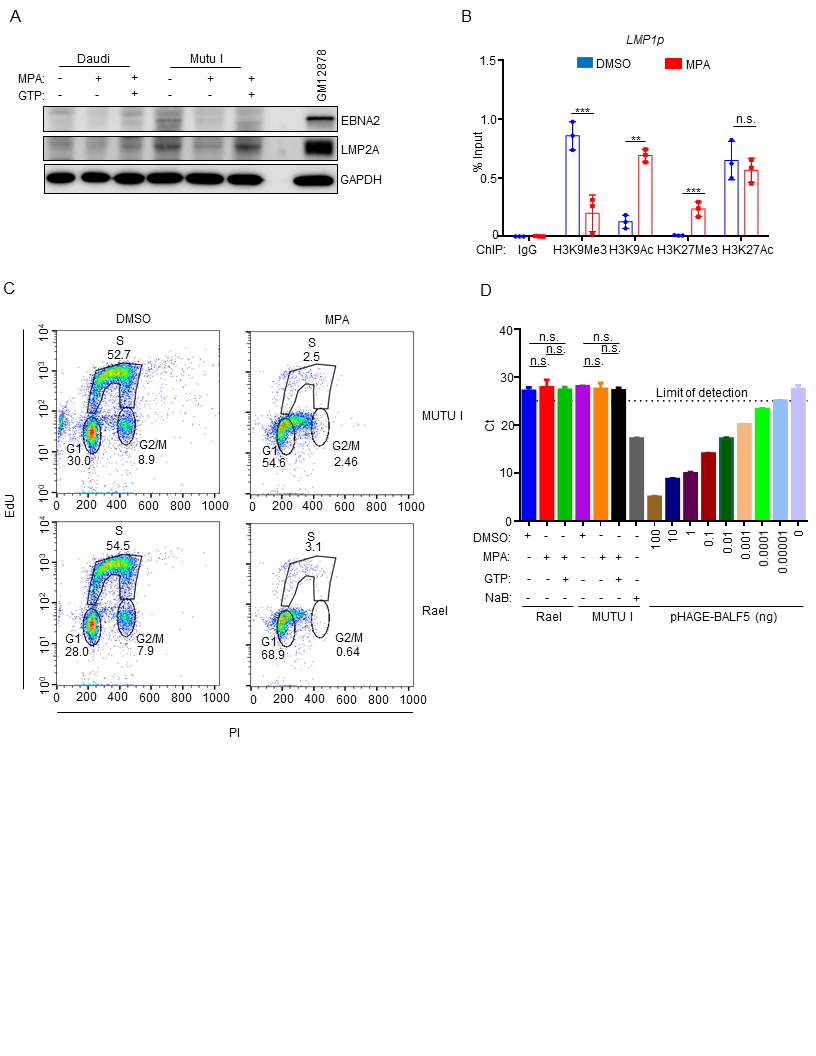

Supplement: S18 Fig — (A) Immunoblot analysis of WCL from Daudi (left) versus MUTU I (right) treated with DMSO, 1 μM MPA ± 100 μM GTP for 24 hours. WCL for latency III GM12878 LCLs was run in the rightmost lane as a positive control for EBNA2 and LMP2A expression. Daudi contain an EBV genomic deletion that knocks out the EBNA2 gene. (B) ChIP-qPCR analysis of Daudi cells treated with DMSO or 1 μM MPA for 72 hours, using the indicated ChIP antibodies and qPCR primers specific for the LMP1p region. (C) FACS analysis of MPA effects on Burkitt cell cycle. Shown are representative FACS plots of 5-ethynyl-2′-deoxyuridine (EdU) vs propidium iodide (PI) levels in MUTU I (top) versus Rael Burkitt cells treated with DMSO or MPA (1 μM) for 48 hours. Gating was constructed using DMSO treated Burkitt cells. Percentages of total cells in each cell cycle phase are shown. Data is representative of n = 3 independent experiments. (D) Analysis of MPA effects on secreted EBV copy number. Shown are the mean ± S.D. values from qPCR analysis using primers shown in Table 2 of DNAse-treated supernatants from Rael or MUTU I cells, which were incubated with DMSO, MPA, GTP and/or the HDAC inhibitor sodium butyrate (NaB) for 96 hours. Shown to the right is the standard curve generated from the indicated amounts of the pHAGE-BALF5 plasmid. The limit of detection was determined by the pHAGE-the BALF5 serial dilution curve. Statistical analysis of Rael and Mutu I supernatants were performed by cross-comparison with the respective DMSO-treated qPCR Ct result. n.s. = not significant. (TIF) [file ppat.1013092.s021.tif]
