# Supplementary material for: Epstein-Barr virus latent membrane protein 1 subverts IMPDH pathways to drive B-cell oncometabolism
Source: PLoS Pathog. 2025 May 14;21(5):e1013092. doi: 10.1371/journal.ppat.1013092 (PMC12169587; doi:10.1371/journal.ppat.1013092)
Supplement: Attachment — Submitted filename: Response to Reviewers 03262025.docx [file ppat.1013092.s023.docx]

**Point-for-Point response.**

**We thank the editor and each of the reviewers for their constructive feedback. Each of the points are responded to below, after the >> symbol.**

**Additional Editor Comments:**
The three reviews all appreciate the importance of the finding and the quality of the experimental data. However, the inability to pinpoint a mechanism reduces enthusiasm and all request additional experiments evaluating mechanism. I think it is likely that you may already have most of this data or can readily obtain it. Reviewer #1 wants to know how established LMP1 induced pathways, NF-kB, Erk, PKC, contribute to this and the contribution of p53 to the induced apoptosis. This is interesting given that the BL cell lines likely have mutant p53. Reviewer #2 is also interested in this and requests assessment of the MPA induced cell cycle arrest. This reviewer has other interesting questions and suggestions although assessment of viral replication is of tangential interest. Reviewer #3 summarizes nicely that "The only significant limitation of this study is that they stop short of defining exactly how LMP1 is upregulating IMPDH2 activity" and would like inclusion of any analyses of cytoophidium.

>>thank you for these important points, each of which are addressed below.

**Reviewers' Comments:**
**Part I - Summary**
**Reviewer #1**: Burton et al have investigated the metabolic reprogramming of B lymphocytes by the Epstein-Barr virus (EBV) oncoprotein LMP1. The study is based on metabolomic analysis of Burkitt’s lymphoma (BL) cell lines with inducible LMP1 expression. The authors focused their analysis on the ability of LMP1 to increase the levels of xanthosine-5-phosphate (XMP), a precursor of guanine nucleotide biosynthesis. Interestingly, LMP1 expression was associated with an increased dependency on the activity of inosine monophosphate dehydrogenases (IMPDH), which catalyze the conversion of inosine monophosphate (IMP) to XMP, the first committed step in guanine nucleotide biosynthesis. More specifically, inhibition of IMPDH by mycophenolic acid (MPA) caused growth arrest of LMP1-negative BL cell lines and apoptosis of LMP1-expressing BL and EBV-transformed lymphoblastoid cell lines (LCL). The dependency of LCL survival on IMPDH was verified by genetic inactivation of the corresponding genes and showed a greater dependency on IMPDH2. Both carboxyl-terminal domains of LMP1 that play a critical role in B cell transformation were involved in XMP increased levels.
This study identifies important elements of B cell metabolic reprogramming that are caused by LMP1 in addition to relevant vulnerabilities that could be exploited for therapeutic purposes. However, it falls short of clarifying important mechanistic aspects of LMP1-mediated upregulation of XMP. LMP1 does not increase the levels of IMPDH1 or IMPDH2 and the authors speculate that LMP1 induces their activity. Nevertheless, they do not provide any direct evidence for LMP1-mediated activation of IMPDH1/2 or a relevant mechanistic explanation. It is known that actively proliferating cells depend on the activity of IMPDH and particularly IMPDH2, which is confirmed in the present study. In the absence of mechanistic data that link LMP1 to IMPDH1/2 activity the report by Burton et al offers limited advancement to the current state of the art.

**>>** We thank the reviewer for their excellent summary and comments.

While IMPDH has certainly been described to have roles in support of cell proliferation, we go well beyond that in this study. For instance, we provide evidence that Latency I Burkitt cells rely on both IMPDH isoforms for growth and survival, whereas latency III lymphoblastoid cells are dependent on IMPDH2. We wish to emphasize that there are significant advances presented here, beyond the observation that IMPDH supports proliferation. We newly identify that IMPDH activity supports survival of LMP1+ B-cells, not previously reported and not simply a proliferative biology phenotype as latency I Burkitt cells grow faster than latency III LCLs. We newly identify XMP as a major metabolite downstream of LMP1 and provide the first metabolomic analyses of LMP1 TES1, TES2 or both. Our study provides the first metabolomic analyses that cross-compare LMP1 TES1 and TES2 roles in metabolome remodeling. We now also provide comprehensive LC/MS analyses of metabolomic remodeling roles of multiple key pathways downstream of LMP1, including on XMP1.

**Reviewer #2**: Burton et al. argue that the Epstein-Barr virus (EBV) latency protein LMP1 both increases activity of inosine monophosphate dehydrogenase (IMPDH) and establishes a dependency of EBV-infected B cells on IMPDH for de novo purine synthesis. The authors begin by inducing LMP1 expression in Burkitt lymphoma Daudi (EBV+) and Akata (EBV-) lines, then analyzing changes in metabolite abundance. They reveal LMP1-dependent increases in purine biosynthesis intermediates (notably xanthosine-5-phosphate), and glutamine. Next, the authors showed a dose-dependent decrease in proliferation of Burkitt lines and lymphoblastoid cell lines (LCLs) after treatment with the IMPDH inhibitor MPA. Additionally, MPA treatment induced a dose-dependent increase in apoptosis in LCLs, which was rescued by GTP addition. Knockout of either IMPDH1 or IMPDH2 decreased growth and increased death of LCLs (with IMPDH2 knockout having a stronger phenotype), while knockout of both IMPDH1 and IMPDH2 was required to stunt growth of Burkitt lymphoma lines, highlighting a heightened dependence of LCLs on individual IMPDH isoforms. In order to test which of the LMP1 cytoplasmic domains were responsible for induction of GTP biosynthesis, the authors conditionally expressed mutant LMP1s in Burkitt lymphoma cells, with either the TES1 or TES2 domain mutated such that it is nonfunctional. Mutating either TES decreased the sensitivity of Burkitt lymphoma lines to IMPDH inhibition, with lines with dual TES mutants being mostly resistant to IMPDH inhibition. This finding, coupled with the finding that MPA was only able to induce Burkitt lymphoma cell death upon conditional LMP1 expression allow the authors to argue that LMP1 is necessary for MPA hypersensitivity. Next, the authors show that TES1 more strongly induces GTP metabolism compared to TES2, as Burkitt lines (Daudi) with TES2 mutants had a more significant increase in abundance of purine metabolites relative to uninduced Burkitt cells, compared to TES1 mutants. Last, the authors show that MPA treatment of Burkitt cell lines was sufficient to increase LMP1 expression, which was associated with a decrease in repressive histone modification and increase in activating histone modifications at LMP1 and EBV lytic gene promoters. Overall, the authors provide a potential explanation for why latency III LCLs are more sensitive to IMPDH perturbation than latency I Burkitt cells.

>> We thank the reviewer for their excellent summary and comments.

**Reviewer #3**: Proliferating B and T lymphocytes are singularly dependent on de novo purine biosynthesis [PMID: 415850] and many pharmacologic agents have been developed targeting this vulnerability. Among them is mycophenolic acid (MPA) an inhibitor of Inosine-5′-monophosphate dehydrogenase (IMPDH) which catalyzes the synthesis of xanthsoine-5-phosphate (XMP), the committed step in de novo guanosine production. MPA selectively impairs lymphocyte proliferation and is widely used as an anti-rejection agent in solid organ transplant recipients. This selectivity arises partly from use of salvage pathway by resting cells and from upregulation of the IMPDH2 gene in proliferating lymphocytes [PMID: 21873529, 1345808].

This manuscript by Burton and colleagues is a metabolomic study that uncovers a key role for the EBV LMP1 oncogene in regulating IMPDH activity. They begin by performing polar metabolite LC/MS profiling in the Daudi and Akata Burkitt lymphoma (BL) cell lines with and without LMP1 expression (Fig 1). They find that XMP levels are increased in both BLs and further show that LMP1 expression heightens sensitivity to MPA (Fig 2) and this effect is attributable to the combined effects of the CTAR1/TES1 and CTAR2/TES2 LMP1 domains (Fig 3). They conduct additional polar metabolite LC/MS profiling in the Daudi and Akata BL using LMP1 mutants to distinguish CTAR1/TES1 and CTAR2/TES2 domain effects. Notably, LMP1 does not alter IMPDH expression levels (Fig S4) and they suggest a likely mechanism: LMP1 promotes formation of IMPDH2 filaments which are known to increase its enzymatic activity [e.g., PMID: 31999252]. Finally, they make the interesting observation that LMP1 expression levels are increased by MPA treatment and provide evidence that this is due to lytic LMP1 expression.

This is a very well-done study that manages to provide interesting insights into effects of LMP1 on MPA sensitivity of B lymphocytes. Their findings suggest that MPA sensitivity is primarily a function of B cell activation rather than proliferation per se. This has obvious clinical implications and is impressive considering the extensive prior literature on the subject. (I’m not sure I share their belief that LMP1 is having effects markedly different than CD40L + BCR crosslinking as argued on lines 310-315 – see minor point below). The only significant limitation of this study is that they stop short of defining exactly how LMP1 is upregulating IMPDH2 activity. I agree that LMP1 induced formation of cytoophidium is a very likely mechanism to explain the observed increase in enzymatic activity despite unchanged IMPDH2 expression levels. Such IMPDH2 filaments can be demonstrated by either EM or IF [PMID: 30555474]. My one suggestion for further experiments is to perform IF (or EM if preferred) to fully evaluate this mechanism (the authors suggest they may have done this experiment already on lines 325-326 and it would be sufficient to include this data as a supplemental figure, provided there is an appropriate positive control for the assay’s ability to detect cytoophidium).

>>We thank the reviewer for their excellent summary and comments. Please see below for specific responses to these important points.

**Part II – Major Issues: Key Experiments Required for Acceptance**
**Reviewer #1:** The authors should determine post-translational changes of IMPDH1 and/or IMPDH2 that depend on LMP1 and activate the enzymes.

>>Thank you for this point. While we agree that this is interesting, it is an open-ended question that we think would best be addressed in a future study. A small handful of phosphorylation sites have been identified on IMPDH 1 and 2, but there could be any number of additional PTMs that regulate IMPDH activity. Considering also that there are two IMPDHs, this would be a major undertaking to use LC/MS approaches to identify these PTMs and to then genetically test the roles of each. There are not presently antibodies available to study any IMPDH modifications. We also wish to note that there are additional levels of IMPDH regulation, including oligomerization/cytoophidium. To confirm that our protocol and antibodies were capable of identifying such structures, we first performed IF on normal oral keratinocytes (NOK) treated with MPA, which induces IMPDH cytoophidium in NOK and in other epithelial cells (PMID 35833994, 16243838). MPA induced MPDH1 and IMPDH2 cytoophidium (new Figure S11 and below).

We then stained LMP1-expressing LCL GM12878 with these same antibodies and performed IFA. As shown in Fig S13 and below, we saw a mixture of diffuse cytoplasmic staining, puncta, and large aggregates for both IMPDH1 and IMPDH2.

We next performed confocal analyses on Daudi and Akata-EBV cells, mock induced or induced for LMP1 for 24 hours (Daudi demonstrated increased purine metabolism and sensitivity to MPA at this timepoint of LMP1 expression). However, we observed IMPDH1 and IMPDH2 cytoophidium in Daudi cells even in the absence of LMP1, and did not observe their levels to be substantially increased by LMP1 expression. To investigate what effect MPA treatment had on these Burkitt cells, we also included an MPA-treated condition. Interestingly, MPA treatment reduced the general staining intensity of the IMPDH aggregates but more often promoted long cytoophidium in treated cells. Representative confocal images are shown below and in new Figure S12.

Daudi

Additional aspects that require further investigation, include the mechanism of MPA-induced apoptosis of LMP1-expressing cells.

>> Thank you for this suggestion. While we found that MPA increased caspase 3/7 activity in latency III cells (**Fig 2G**) and that MPA-induced Annexin V positivity in latency III B cells (**Fig 2F**), we further investigated the cell death pathway. Interestingly, the pan-caspase inhibitor Z-VAD-FMK did not block MPA-induced cell death. The necroptosis inhibitor Necrostatin-1s also failed to rescue MPA-inducd cell death (new Figures S7-8 and summarized below). Therefore, consistent with prior reports that MPA induces necrosis (but not necroptosis) in activated lymphocytes (PMID 19017951), we changed the text to reflect these findings and to suggest that MPA may induce necrosis in LMP1+ B cells.

For example, the authors should investigate the role of p53 in MPA-induced apoptosis of LMP1-expressing cells.

>>We thank the reviewer for this suggestion. We observed MPA-induced cell death of latency-III lymphoblastoid cell lines, latency III Burkitt cells and LMP1+ Burkitt cells. We note that many Burkitt cells have inactivating p53 mutations (PMID 1915267) while EBNA3C dampens p53 signaling in LCLs, though not completely (PMID 19144715, 25691063). To test potential p53 roles in MPA-induced cell death of LMP1+ cells, we used the small molecule p53 inhibitor pifithrin-α as well as CRISPR p53 KO in two LCL models. As shown in Fig S4-S6 (and below), p53 inhibition did not rescue cell death, suggesting that MPA-induced death does not dependent on p53. This has been elaborated on in the text.

**Figure S4**

**FigureS5**

**Figure S6**

In addition, it would be informative to determine the potential role of NF-kappaB and/or MAPK activation by LMP1 in XMP upregulation

>>Thank you for this suggestion. Since our data suggests TES1 and TES2 roles in purine biosynthesis and MPA sensitivity, we conducted broad metabolomic analyses, using well-characterized small molecule inhibitors at published doses (it would not be feasible to do these analyses with CRISPR, as knockouts of multiple LMP1 pathways would induce cell death, but metabolomic analyses can be performed at early timepoints of chemical inhibition, where cells are still viable). We therefore conditionally expressed LMP1 in Burkitt cells in the presence of DMSO vehicle or inhibitor of PKC, JNK, ERK, p38 or IKKβ. LC/MS metabolomic analyses revealed major effects of each of these inhibitors on the metabolomes of LMP1+ cells. Notably, a many purine nucleotide pathway metabolites were downregulated by staurosporine PKC inhibition. Importantly, inhibition of any of these pathways impaired LMP1 upregulation of XMP. Our analyses therefore highlight roles of multiple pathways downstream of LMP1 in XMP metabolism, which we look forward to investigating in depth in a future study. These new data are presented in new **Fig 6 and below**.

**Reviewer #2**: 1. BrdU/7-AAD experiment to test whether the MPA-mediated growth inhibition in Burkitt cell lines is at the G1/S phase where one might expect.

>>Thank you for the suggestion. We used EdU vs PI staining to investigate MPA-mediated cell cycle effects on Burkitt lymphoma cells. As anticipated by the reviewer, MPA caused G1 arrest in both MUTU I and Rael Burkitt cells (shown below and in new **Figure S17C**).

2. Figure S2E: show ATM/ATR phosphorylation in MPA-only treated LCLs. Add a positive control (e.g. lysate from cells treated with ionizing radiation).

>>Thank you for catching this. The samples have been relabeled, as we indeed had MPA-only LCLs. Interestingly, Latency III MUTU cells have basal ATR and ATM phosphorylation, now shown in (**Fig. S2E**). Rather than ionizing radiation, we used etoposide to induce DNA damage. Etoposide highly induced ATM and ATR in MUTU I and III cells, but interestingly not in GM12878 LCLs. We note that these results were similar to those produced by MPA.

3. Figure 4/5: test whether there are any growth differences between TES1 and TES2 mutants in the conditionally-expressing Burkitt cells to see whether the increased effect on purine metabolism by TES1 results in TES1 mutants growing slower.

>>Thank you for the suggestion. We performed CFSE dye-dilution assays on Daudi Burkitt cells conditionally expressing WT, TES1 or TES2 mutant LMP1. We did not see any significant differences in growth (**Fig 4G, below**), likely because Burkitt cells already grow rapidly. Therefore, differences in growth rates do not appear to account for LMP1-driven increases in purine metabolism.

4. Figure 6: measure extracellular EBV genome copy number in Burkitt cells after MPA treatment to see whether this lytic reactivation is productive.

>>Thank you for the suggestion. We treated MUTU I and Rael Burkitt cells with MPA, with GTP rescue as a control to quench MPA-driven lytic gene induction. We used sodium butyrate as a positive control. qPCR analysis on DNAse treated supernatants did not detect MPA-driven secretion of encapsidated genomes (**Fig S17D and shown below**). Therefore, MPA induces abortive lytic replication.

**Reviewer #3**: 1) An experiment examining whether LMP1 induces formation of IMPDH2 filaments (cytoophidium) should be done (and, if results are negative, a positive control validating the assay is essential).

Thank you for this suggestion. We performed immunofluorescence assays on Akata and Daudi cells with conditional LMP1 expression, which demonstrated LMP1-driven increase in purine metabolism and sensitization to MPA-triggered cell death. As a + control, we performed confocal analyses on normal oral keratinocytes treated with MPA, as MPA has been published to trigger cytoophidium in multiple epithelial cell contexts (PMID 35833994, 16243838). MPA triggered IMPDH1 and to a slightly lesser extent IMPDH2 cytoophidium **(Fig. S11 and below).**

We then stained LMP1-expressing LCL GM12878 with these same antibodies and performed IFA. As shown in Fig S13 and below, we saw a mixture of diffuse cytoplasmic staining, puncta, and large aggregates for both IMPDH1 and IMPDH2.

We next performed confocal analyses on Daudi and Akata-EBV cells, mock induced or induced for LMP1 for 24 hours (Daudi demonstrated increased purine metabolism and sensitivity to MPA at this timepoint of LMP1 expression). However, we observed IMPDH1 and IMPDH2 cytoophidium in Daudi cells even in the absence of LMP1, and did not observe their levels to be substantially increased by LMP1 expression. To investigate what effect MPA treatment had on these Burkitt cells, we also included an MPA-treated condition. Interestingly, MPA treatment reduced the general staining intensity of the IMPDH aggregates but more often promoted long cytoophidium in treated cells. Representative confocal images are shown below and in new Figure S12.

Daudi pLX LMP1 cells

However, the cytoophidium number or morphology did not change substantially with LMP1 expression (**Fig S12 and above**), suggesting that alternative mechanisms underlie LMP1 driven increase in XMP.

**Part III – Minor Issues: Editorial and Data Presentation Modifications**

Reviewer #1: Does doxycycline alone affect XMP levels?

>>Thank you for the question. We wish to emphasize that we used a low level of doxycycline (250ng/mL) and only for 24 hours. Effects of doxycycline on mitochondrial metabolism are observed over longer time periods and at higher levels of doxycycline in published studies. Furthermore, levels of XMP clearly changed between WT, TES1, and TES2 mutant conditions, all of which were treated with dox (**Fig. 5**). This suggests that the effects we see cannot be explained by doxycycline alone.

In Figure 1E what does Try stand for?

> Thanks for the comment. Try should have been Tyr (for tyrosine). We fixed this typo.

Lines172-173: The sentence “Unexpectedly, MPA lowered …levels” should be extended as follows “Unexpectedly, MPA lowered ATM and ATR phosphorylation levels to a greater extent in latency III compared to latency I B cells”

>Thanks, we corrected this.

Figure S4A and B: correct “IMDPH”
>>Thank you, we corrected this typo.

The text must be checked for typos i.e. line 128 xanthsoine, in line 152 the word “inhibition” is repeated twice.

>>Thanks for catching these typos, which we corrected.

The name initials in funding details are wrong.

>>Thank you, we corrected the name initials.

Reviewer #2:

1. Figure 2B: missing asterisks for significance.

>> Thanks, we corrected this mistake.

2. Figure 4E: the green color of bar plots is not consistent throughout the graph.

>> Thanks, this has been adjusted.

3. Figure 4E-F: the color key and +/- treatment indicators below the graphs do not correspond with each other. Green color says that both MPA and GTP are added, while the +/- indication says that only GTP is added.

>> Thanks, this has been corrected.

4. Line 253: typo (“purine synthesis to support of GTP abundance”)
>> Thank you, this typo has been corrected.

5. Line 272: typo (“neither LMP1 or lytic cycle”)
>> Thank you, this has been corrected.

6. Line 334: typo (“CRIPSR”)

>> Thank you, corrected!

Reviewer #3:

1) Lines 22-24, 48-50, 80-81: I don’t share the view that LMP1 is sufficient to transform B cells.

We know this is not true for human B cells [PMID: 38169736] and the murine studies cited to support that this is true for murine B cells do not exclude the possibility that somatic mutations are required to cooperate with LMP1 to induce transformation in that model.
>>Thank you for this feedback. We were referring to fatal lymphoproliferative disease that rapidly arises in LMP1 transgenic mice with T-cell suppression (PMID 22341446). However, we agree that it is not possible to exclude secondary mutations in B-cell transformation, and have therefore modified the text as suggested.

2) Lines 69-79 - the EBERs, BARTs and BART-miRs need to be added to this.
>>Thank you, we added these to the introduction.

3) lines 128-131 – “XMP . . . was the most highly LMP1 upregulated metabolite . . .” Based on what? GMP appears to be more upregulated in Daudi (Fig 1B), hypoxanthine more upregulated in Akata (Fig 1C).
>>Thank you, we changed this statement to imply that XMP is one of the most highly upregulated metabolites in common between both Daudi and Akata Burkitt lymphoma cells expressing LMP1.

4) Figure 1G is excellent, but please ensure that the same names are used as in Figs 1BCF. For example, I believe that XMP and Xanthosine-5-P refer to the same molecule. Please pick one name and stick to it throughout for all metabolites.
>>Thanks, we now refer to it as XMP throughout.

5) Why was Fas assayed in Daudi (Fig 1B) and ICAM-1 used for Akata (1D)? Use of the same assay or, better yet, showing both would be more convincing.
>>Thanks for the opportunity to clarify. For unclear reasons, Daudi express elevated levels of ICAM-1, whereas Akata instead have high basal Fas levels. For these reasons, we have used Fas in Daudi and ICAM-1 in Akata in prior published studies, such as PMID 38009935. We now clarified this point in the text.

6) Since the signaling tail of LMP1 is on the C-terminus, it would be preferable if Fig7 were re-drawn such that the TMs are to the left of TES1/TES2 domains (I realize this is a pretty minor point)

>>Thank you, we made the suggested change (**Fig. 8. See below**).

7) In the results it is mentioned that Daudi LMP1 migrates at a different MW, but Fig 6B does not appear to show any convincing amount of LMP1 expression in these cells. Is LMP1 cropped out of the panel? If not, then there does not appear to be any credible induction of LMP1 in Daudi cells and the ChIPs in Fig 6C-F are uninformative and need to be done in Mutu I cells if any insights into LMP1 induction by MPA is to be gained.

>> Thank you for the opportunity to clarify this point. Indeed, ChIPs for the main text figure were performed in MUTU I, which we now properly state in the Figure legend (thanks for catching the typo). Of note, prior Fig. 6 is now Fig. 7 in the revised manuscript. We removed the LMP1 ChIP performed in Daudi, which was previously shown in the supplement.

8) A previous study examined donor-specific genetic determinants of MPA susceptibility among LCLs [PMID: 21396482] – if they think that any of these intersect with metabolism or LMP1 the authors should consider adding this to their discussion.
>> Thx for this suggestion, which we now comment on in our discussion.

9) lines 310-315 – this may be true, but may also be a limitation of modeling GC reactions via 24 hour stimuli. The finding of IMPDH2 filaments in GC reactions [PMID: 30555474] would argue its activity is markedly upregulated by physiologic lymphocyte activation.
>>Thank you, we now include this point in the discussion.

10) lines 343-344 – one should not conclude that EBNA3C attenuates p53 function in latency III based on studies performed in osteosarcoma cells when the Luftig lab has clearly demonstrated that LCLs (an accepted latency III model) have intact p53 function despite EBNA3C expression.

>>Thanks, we also found that MDM2 KO was lethal to GM12878 but not P3HR1 Burkitt cells in our published CRISPR screen (PMID 28494239). While we generally agree with this point, it remains plausible that EBNA3C dampens p53 without altogether inhibiting it. In this scenario, MDM2 inhibition highly upregulates p53 protein levels and overcomes this threshold.
